# Physically Consistent Self-Diffusion Coefficient Calculation with Molecular Dynamics and Symbolic Regression

**DOI:** 10.3390/ijms26146748

**Published:** 2025-07-14

**Authors:** Dimitrios Angelis, Chrysostomos Georgakopoulos, Filippos Sofos, Theodoros E. Karakasidis

**Affiliations:** Condensed Matter Physics Laboratory, Department of Physics, University of Thessaly, 35100 Lamia, Greece; dimangelis@uth.gr (D.A.); chgeorgak@uth.gr (C.G.); thkarak@uth.gr (T.E.K.)

**Keywords:** symbolic regression, diffusion coefficient, molecular dynamics, molecular fluids

## Abstract

Machine Learning methods are exploited to extract a universal approach for self-diffusion coefficient calculation in molecular fluids. Analytical expressions are derived through symbolic regression for fluids both in bulk and confined nanochannels. The symbolic regression framework is trained on simulation data from molecular dynamics and correlates the values of the self-diffusion coefficients with macroscopic properties, such as density, temperature, and the width of confinement. New expressions are derived for nine different molecular fluids, while an all-fluid universal equation is extracted to capture molecular behavior as well. In such a way, a highly computationally demanding property is predicted by easy-to-define macroscopic parameters, bypassing traditional numerical methods based on mean squared displacement and autocorrelation functions at the atomistic level. To achieve generalizability and interpretability, simple symbolic expressions are selected from a pool of genetic programming-derived equations. The obtained expressions present physical consistency, and they are discussed in terms of explainability. The accurate prediction of the self-diffusion coefficient both in bulk and confined systems is important for advancing the fundamental understanding of fluid behavior and leading the design of nanoscale confinement devices containing real molecular fluids.

## 1. Introduction

The theoretical and experimental investigation of systems that consist of atoms or molecules in constant motion is essential to understanding their behavior under various conditions or states (bulk or confined). However, barriers related to time-consuming and expensive experiments and system investigation under extreme temperature or pressure conditions usually emerge. As an alternative, computer simulations are employed to create a connection between models, theoretical predictions, and experimental results [1], becoming a *virtual laboratory* [2] that provides accurate results. To this end, diverse modeling techniques that employ advanced statistical mechanics methods and complex calculations at all scales are employed, such as molecular dynamics (MD) [3], Monte Carlo (MC) [4], hybrid MD and first-principles [5], quantum-based [6], and molecular-continuum techniques [7], to mention a few.

Molecular dynamics simulation integrates the classical equations of motion to deliver time-resolved atomistic trajectories, enabling the direct calculation of both static and dynamic properties, posing as the primary computational method for condensed matter systems from the atomic scale to microscale [8]. Its performance is strongly based on the employed interaction potential between particles, with the Lennard-Jones (LJ) potential being the common choice for simplicity and fast execution [9,10,11]. Consequently, valuable information is generated at the microscopic scale (particle position, velocity, trajectory, etc.), which can be converted to observable macroscopic variables such as temperature, pressure, and density. Constantly increasing computing power has rendered MD simulations a convenient tool for the research of nanoscale fluid flow [12].

Data obtained via MD simulations can then be analyzed by statistical algorithms through exploiting Machine Learning (ML) methods, either as a post-processing step or in a hybrid manner [13]. These algorithms learn from *experience* and extract useful expressions and hidden correlations [14]. Machine Learning has made an emphatic impact on the physical sciences, with applications ranging from statistical physics to chemistry and materials, using classification, clustering, and regression techniques, each one with different objectives [15,16,17]. Nonetheless, while ML is highly successful in producing accurate predictions, it suffers from significant interpretability limitations.

Accordingly, symbolic regression (SR) has emerged [18]. This refers to a supervised ML technique that exploits mathematical operators or functions in order to find a simple and accurate model that best fits a given dataset, derived either from simulations or experiments [19]. In such a way, SR is focused on discovering the relationship between the input parameters and the target property, suggesting an accurate and extensible relation to employ in system design and justification [20]. Some example applications include materials science and engineering [21,22], hydrodynamics [23,24], energy applications [25], and environmental sciences [26].

This paper focuses on the calculation of the diffusion coefficient, *D*, one of the main fluid transport properties and a key process in mass transfer. Diffusion depends on how fluids respond to changes in temperature or density, and to the structures that confine them. Ranging from micro- to macroscopic methodologies, various techniques have been developed over the years to estimate *D*. Among them, particle methods at the atomic scale (e.g., MD) have been established as the most suitable tool for their calculation, mainly due to their ability to approach the solution by a physics-driven methodology and achieve high accuracy [27,28,29]. In MD, particle positions, velocities, and trajectories are extracted during the simulations and used in statistical mechanics equations to derive time-dependent properties at equilibrium or non-equilibrium conditions.

Moreover, empirical relations [30] have been employed, along with recent ML methods based on simulation data [10,31]. It has been reported that *D* is linearly dependent on temperature [11,28,31,32,33,34] as higher temperatures enhance thermal movement and promote diffusion. In terms of density, ρ, an inversely proportional relationship between *D* and ρ is also observed. This behavior is consistent with the physical behavior of the system since low-density fluids have shown higher *D* values. In the case of confined systems (i.e., nanochannels), the pore size is also a key parameter. For instance, fluid diffusion coefficients have been found to increase with channel width [35,36,37], while *D* approaches its bulk value as the channel width increases beyond a certain point [37,38]. It has also been reported that, for large pore sizes, *D* may even exceed the bulk values [39].

Here, an SR framework is implemented, where the extracted equations provide a valid physical explanation. The reduced self-diffusion coefficient, $D^{*}$, of nine molecular fluids in the liquid state is provided with microscopic accuracy by employing reduced macroscale properties, such as temperature $T^{*}$ and density $\rho^{*}$ at bulk. Moreover, $D^{*}$ is also given for confined nanochannels, where the pore size, $H^{*}$, is an additional input parameter. The training dataset comes from MD simulations, taken from Leverant et al. [35] and enriched by our own simulations, while output expressions are derived both for each molecular fluid and as a universal expression that covers all fluids. To our knowledge, this is the first attempt to express the self-diffusion coefficient with a universal equation that applies over a wide range of molecular fluids.

The derived equations are of low complexity and high accuracy, depending only on three macroscopic (reduced) variables, i.e., $T^{*}$, $\rho^{*}$, and $H^{*}$ (where applied). Our investigation abides by the following directions:

- Accuracy: Evaluation with the appropriate statistical measures.
- Complexity: Avoid complex mathematical expressions.
- Equation recurrence: Focus on repeating patterns.
- Data analysis: Focus on the physical interpretation of the result, taking into account the correlation of the system variables.

## 2. Results and Discussion

The adopted methodology behind the selection of final expressions is outlined in Section 2.1. In Section 2.2, the suggested symbolic expressions along with their accuracy measures are presented, followed by a detailed comparison between the SR results and the MD database.

### 2.1. Expression Selection Methodology

By training the SR model with data derived from MD simulations and expressing the resulting correlations in symbolic form, we facilitate more efficient bridging across scales, achieving generalization ability and interpretability of the final expressions. While the MD framework runs on atomic-scale detail, considering interatomic forces, particle positions, and velocities, the self-diffusion coefficients are connected to $\rho^{*}$, $T^{*}$, and $H^{*}$, which are macroscale properties. The training data correspond to 80% of the available datapoints, and the remaining 20% comprises the validation set.

A multi-stage approach is implemented at various stages of the procedure. The accuracy of the obtained expressions is evaluated in terms of the coefficient of determination ($R^{2}$), which provides a measure of the model’s overall fit and explanatory power, and the average absolute deviation (AAD), which quantifies the average deviation of predictions from their true values, given by [11,40]

(1) $$R^{2}=1-\frac{\sum_{i=1}^{n}{\left(y_{i}-{\hat{y}}_{i}\right)}^{2}}{\sum_{i=1}^{n}{\left(y_{i}-\bar{y}\right)}^{2}},$$

(2) $$AAD=\frac{1}{n}\sum_{i=1}^{n}\left|y_{i}-{\hat{y}}_{i}\right|,$$

where $y_{i}$ and ${\hat{y}}_{i}$ are the MD-generated values and the SR predicted value for the *i*th component, respectively, $\bar{y}$ is the average MD data value, and *n* the total number of datapoints. Accuracy is closely connected to expression complexity, focusing on simple expressions, which may be more easily mapped to physical laws, while addressing potential overfitting issues [41,42].

A number of GP-based iterative runs are performed, each one at a different random seed, to mitigate the impact of randomness of the results on the form of the output expression. The repetition of a certain expression on such a random procedure is an indication of capturing core behaviors on the dataset and requires further attention [11].

The SR framework runs independently for each molecular fluid, resulting in dedicated symbolic expressions, along with an all-molecule universal approach that accounts for all fluids. Considering that the reduced parameters employed in the MD simulations (Equation (15)) embed ϵ, σ, and *m* in the calculation of the reduced quantities, their effect is implicitly incorporated into the final values of $\rho^{*}$, $T^{*}$, $H^{*}$, and $D^{*}$, thereby indirectly affecting the model output.

### 2.2. Comparison with Simulation Data

#### 2.2.1. Bulk Fluids

Starting from the bulk dataset, the derived SR expressions are in the form

(3) $$D_{SR}^{*}=\alpha_{1}\;\frac{T^{*\alpha_{2}}}{\rho^{*\alpha_{3}}}-\alpha_{4},$$

where the values of the parameters $\alpha_{i}$ (where i=1,…,4) differ for each molecular fluid. Their approximate values are listed in Table 1.

**Figure 1:**
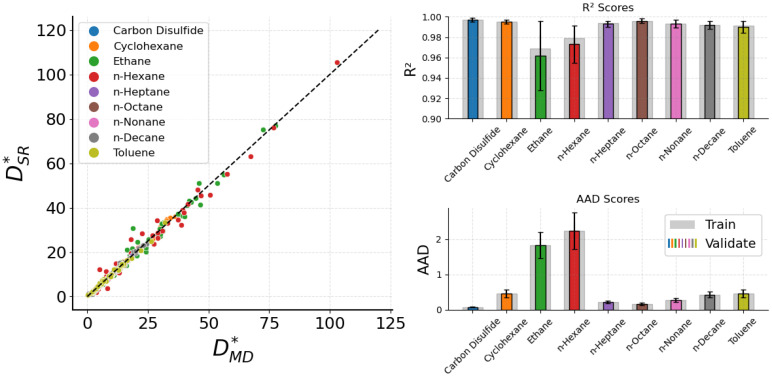
A collective identity plot between $D_{SR}^{*}$ from Equation (3) and $D_{MD}^{*}$ of the bulk case, with barplots presenting the $R^{2}$ and AAD scores for the training and validation datasets.

This obtained form reflects the expected physical behavior, where $D^{*}$ is inversely proportional to $\rho^{*}$ and proportional to $T^{*}$, which follows the qualitative trend found during the preprocessing analysis in Section 3.3. Regarding the accuracy of the expressions, a collective identity plot of the nine dedicated expressions along with their $R^{2}$ and AAD values is presented in Figure 1. To improve the robustness of the results, a repeated k-fold cross-validation [43] has been performed, and the average $R^{2}$ and AAD values are shown, with the error bars corresponding to the standard deviations. Colored bars represent the validation dataset metrics, while training bars are shown in light gray color. Overall, the molecular fluid dedicated expressions exhibit fine accuracy, achieving in most cases $R^{2}$ values higher than 0.98 and AAD lower than 0.5. Small deviations occur only for the ethane and n-hexane fluids, where, although their $R^{2}$ values are higher than 0.96, the respective AAD is notably higher than every other fluid. From Table 2, we observe that $D^{*}$ for ethane ranges from 6.475 to 77.694 and for n-hexane from 3.621 to 102.942, both significantly wider compared to the other fluids. This has a profound effect on the statistical accuracy of the predictions for these two fluids. To increase the accuracy, if unsatisfactory, we would need more datapoints from MD simulations.

Next, a universal expression is proposed by the SR model, considering all nine datasets as one, which captures the behavior of all molecular fluids as follows:

(4) $$D_{SR}^{*}=\frac{48.34\;T^{*0.97}-9.90}{\rho^{*}}-34.12\;T^{*1.13}.$$

**Figure 2:**
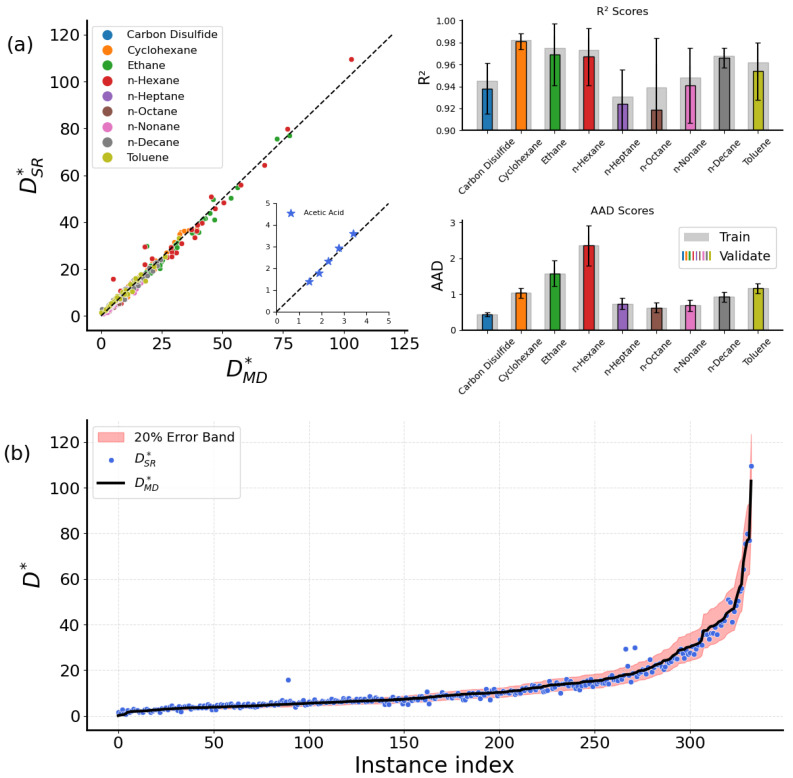
(**a**) A collective identity plot between $D_{SR}^{*}$ from Equation (4) and $D_{MD}^{*}$ of the bulk case, with barplots presenting the $R^{2}$ and AAD scores for the training and validation datasets. The inset with the star points presents the fit of a new fluid (not in the original dataset), acetic acid, on Equation (4). (**b**) $D_{SR}^{*}$ and $D_{MD}^{*}$ comparison. Values are sorted for presentation reasons.

This expression also captures the dependence of $D^{*}$ with $\rho^{*}$ and $T^{*}$, similar to Equation (3). Since all-fluid behavior is captured, it is expected that more terms would contribute in a more complex manner. Complexity is higher as a correction term appears on the fraction $\frac{T^{*}}{\rho^{*}}$, and a negative temperature-dependent factor replaces the previously constant term. Liquids at bulk state face confinement effects, which make them spend more time oscillating around their local sites of residence before they can diffuse [44]. As the temperature increases, the fluid obtains the essential energy needed to diffuse through jump events. However, in parallel, this leads to greater vibrational amplitudes, eventually blocking these jumps, and this is reflected by the negative temperature term that appears. Regarding Equation (4), prediction accuracy is fine when applied to each molecular fluid, as also seen for the dedicated expressions. The results are shown for the identity and accuracy plots in Figure 2a. The $bR^{2}$ and AAD values are slightly lower than those of the dedicated expressions, indicating overall agreement between the two approaches. We have also validated Equation (4) on an unseen fluid from [35], acetic acid. The new datapoints (i.e., stars) fit on the regression line, suggesting fine accuracy regarding the predictions (see the inset in Figure 2a).

In addition, Figure 2b compares all the available $D^{*}$ values from both MD and SR. Most $D_{SR}^{*}$ prediction values fall within an 20% error band. However, deviations exist for lower $D_{SR}^{*}$ values. In light of the above, the all-molecule universal expression of Equation (4) can serve as an alternative to the dedicated ones, offering greater generalization capabilities while simultaneously achieving results comparable to those of the dedicated expressions.

**Figure 3:**
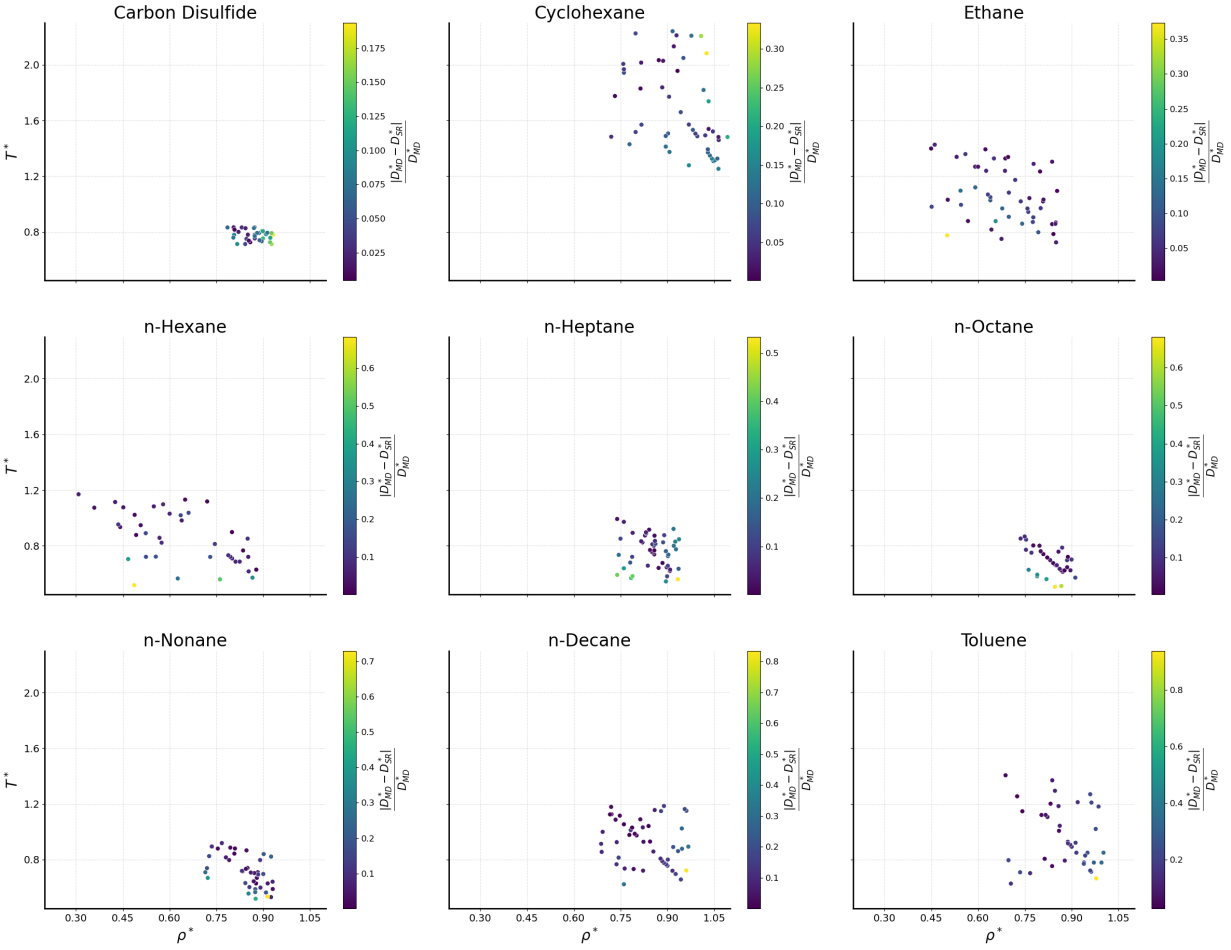
Self-diffusion coefficients in the ($\rho^{*}$-$T^{*}$) phase space in the form of relative absolute error, $|D_{MD}^{*}-D_{SR}^{*}|\;/\;D_{MD}^{*}$, for the bulk fluids.

The prediction performance of the bulk fluids is further investigated in Figure 3 across the phase space ($\rho^{*}-T^{*}$). Datapoints color accounts for the relative absolute error $|D_{MD}^{*}-D_{SR}^{*}|\;/\;D_{MD}^{*}$ with respect to MD data. It seems that the all-fluid universal SR expression Equation (4) effectively captures the MD behavior. Although certain datapoints for specific fluids show differences between the predicted and simulated values, these deviations do not exhibit a consistent pattern with respect to their $\rho^{*}$ or $T^{*}$ values.

#### 2.2.2. Fluids in Nanochannels

We focus next on extracting a universal expression for molecular fluids inside nanochannels. No dedicated expressions are derived for each molecular fluid inside the nanochannel since one would have to take into account the interaction potential between the walls and the fluids and account for various wall materials. The universal expression is derived by further incorporating $H^{*}$ in the calculations as

(5) $$D_{SR}^{*}=\frac{2.29\;T^{*0.98}\;\log\left(H^{*}\right)+62.80\;T^{*0.53}}{\rho^{*0.77}}-78.81\;T^{*0.35}+11.04$$

In this case, a more complex form is obtained compared to Equations (3) and (4). The $T^{*}$ term follows a structure similar to the universal bulk expression of Equation (4) as it is incorporated both in the fraction and as a correction factor. The parameter $H^{*}$, on the other hand, appears in the numerator of the fraction as part of a logarithmic function. In physical terms, as $H^{*}$ increases, $D^{*}$ approaches its bulk value, while $D^{*}$ decreases in case of strong confinement, i.e., for small $H^{*}$. For certain values of $H^{*}$ ($H^{*}=\frac{H}{\sigma }$), the logarithmic term becomes negative, indicating a negative contribution. This behavior can be attributed to molecular confinement effects that arise when the channel width approaches the molecular diameter ($H^{*}<1$), leading to restricted molecular motion and, consequently, a partial decrease in diffusion despite the increase in $T^{*}$. There is also a negative temperature term ($-78.81\;T^{*0.35})$ as in the bulk case (Equation (4)), which leads to diffusion decrease due to enhanced intermolecular interactions at higher temperatures, as discussed in Section 2.2.1.

**Figure 4:**
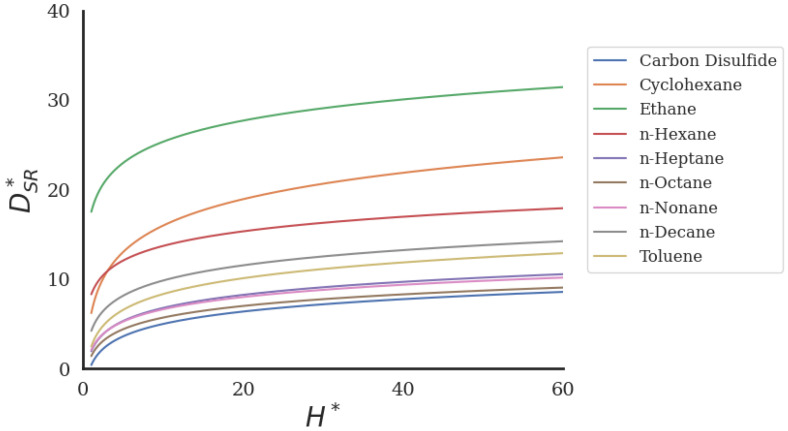
The SR-derived self-diffusion coefficient $D_{SR}^{*}$ from Equation (5) vs. $H^{*}$ for nine molecular fluids inside nanochannels.

To investigate this behavior, Figure 4 presents the values of $D_{SR}^{*}$ vs. $H^{*}$ when $\rho^{*}$ and $T^{*}$ are kept constant at the corresponding mean values of the dataset. The expression yields $D^{*}$ values for which the rate of increase progressively decreases, with $D^{*}$ approaching a nearly constant value. Therefore, the expression in Equation (5) captures the expected physical behavior and is consistent with well-established MD results reported in Giannakopoulos et al. [37] for pure LJ fluids.

**Figure 5:**
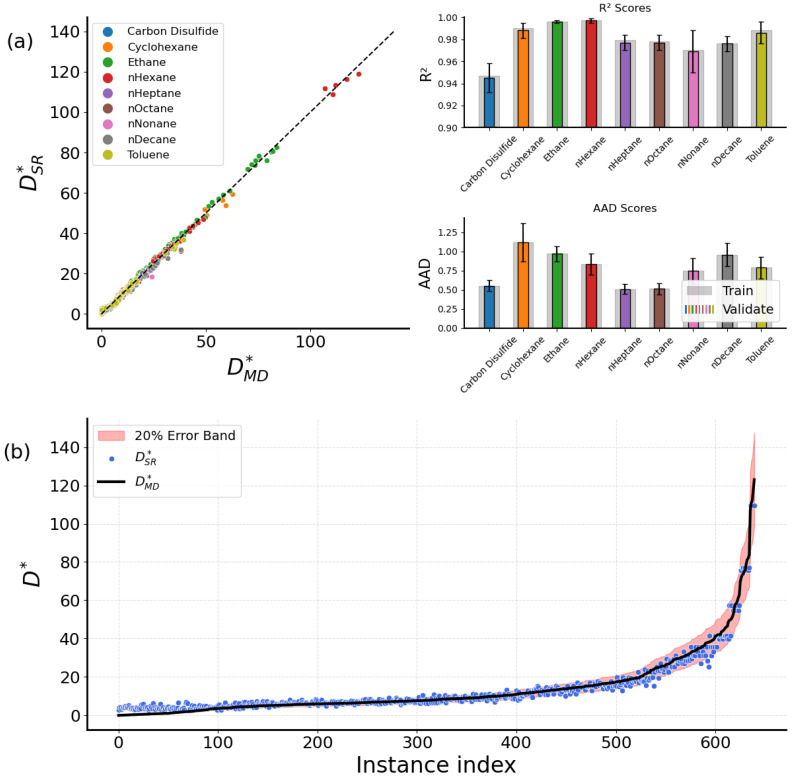
(**a**) A collective identity plot between $D_{SR}^{*}$ from Equation (5) and $D_{MD}^{*}$ of the nanochannels case, with barplots presenting the $R^{2}$ and AAD scores for the training and validation datasets. (**b**) $D_{SR}^{*}$ and $D_{MD}^{*}$ comparison. Values are sorted for presentation reasons.

The identity plot and the performance measures are depicted in Figure 5a. Overall, the expression shows fine predictive performance as the majority of $R^{2}$ values are above 0.96 and all AAD values are below 1.0. On the other hand, Figure 5b has revealed limitations of the symbolic expressions that were not obvious from the respective identity plot. The universal expression for the nanochannels does not fit well on MD data for lower values of $D^{*}$. In contrast, for higher $D^{*}$ values, the model performs well as most predictions fall within a 20% error.

**Figure 6:**
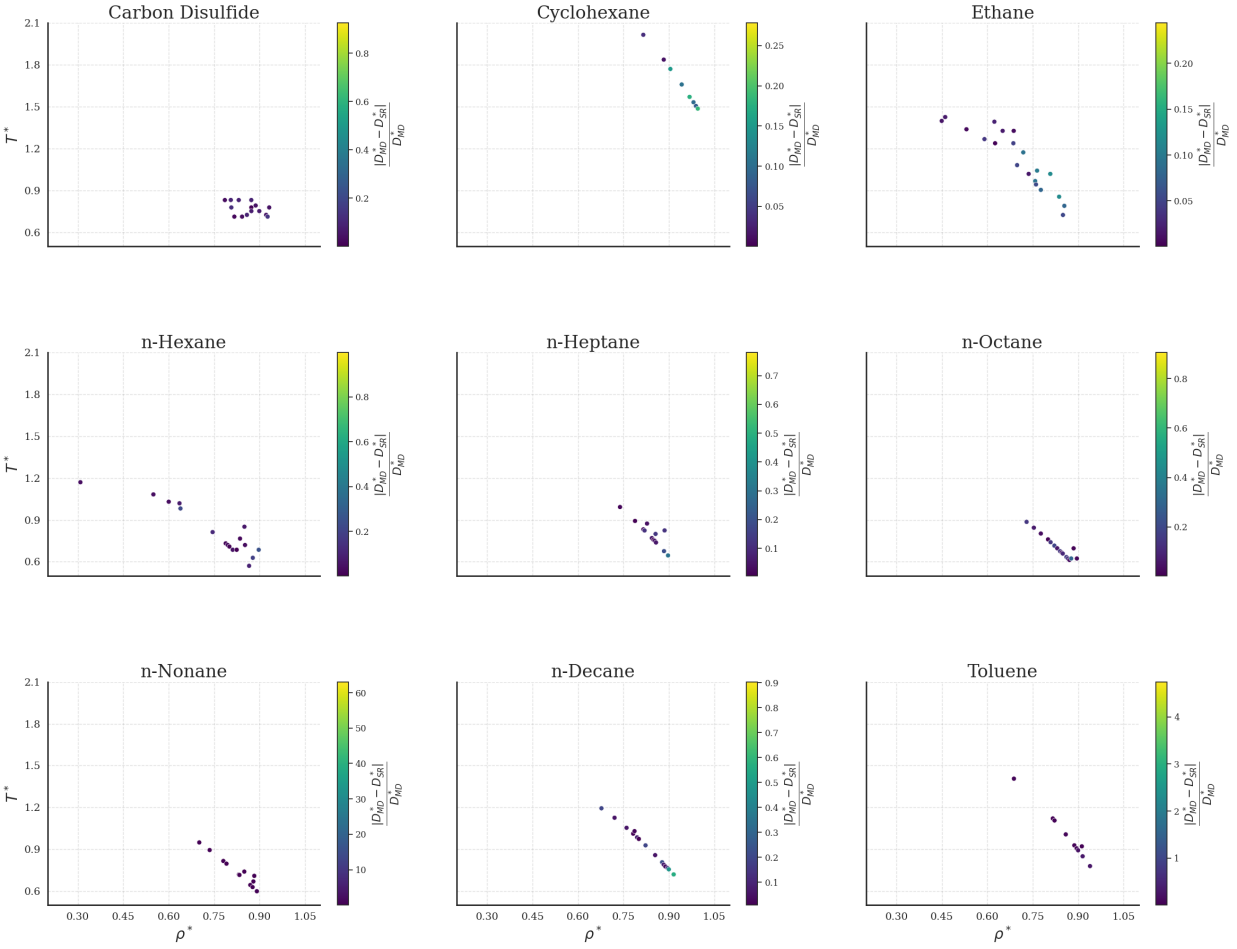
Self-diffusion coefficients in the ($\rho^{*}$-$T^{*}$) phase space in the form of relative absolute error, $|D_{MD}^{*}-D_{SR}^{*}|\;/\;D_{MD}^{*}$, for the fluids inside nanochannels.

**Figure 7:**
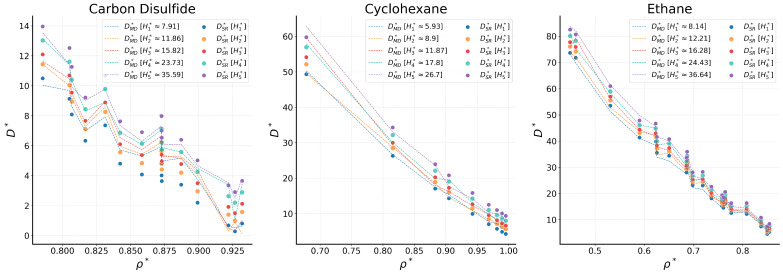

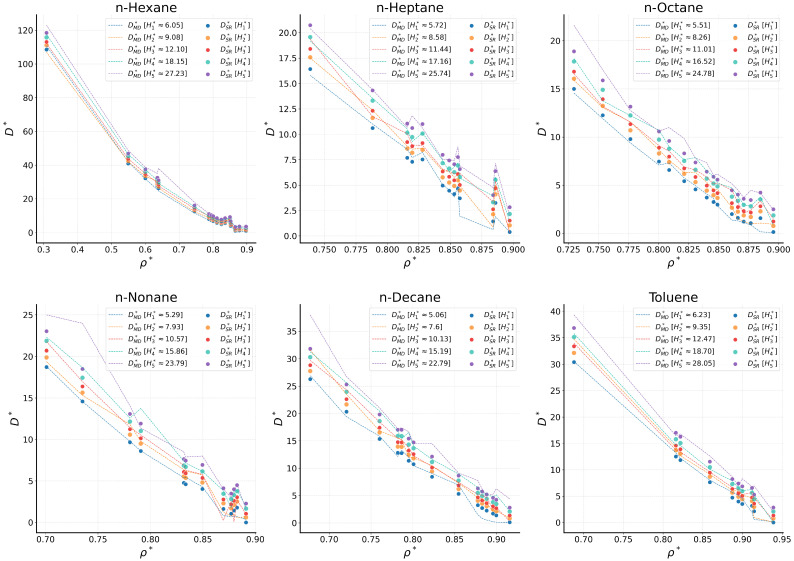
Comparison of MD and SR values of $D^{*}$ vs. $\rho^{*}$ for various $H^{*}$ in nanochannels.

The error phase space is given in Figure 6. As with the bulk counterpart, no clear pattern can be distinguished with respect to the variations in $\rho^{*}$ and $T^{*}$. However, the phase plots exhibit irregularities that are not readily apparent due to overlapping datapoints, which conceal the deviations. For further analysis, we extend our investigation to the $H^{*}$ values as they play a key role in the nanoconfined behavior [38]. Figure 7 shows $\rho^{*}$ vs. $D^{*}$ plots, where $D^{*}$ is calculated for five different $H^{*}$ values. Lineplots correspond to MD data, and scatterplots refer to $D_{SR}^{*}$ from Equation (5).

General agreement between the MD and SR values is observed for most cases, following the trend shown in Figure 5. Nonetheless, deviations exist. For instance, for carbon disulfide, significant deviations appear for the narrower nanochannels (small $H^{*}$) and for moderate to low values of $D^{*}$. These deviations are less apparent for densities up to 0.82 but become increasingly evident as the fluid density increases. Conversely, the symbolic expression exhibits limitations in accurately capturing $D^{*}$ values at higher nanochannel widths for n-octane, n-nonane, n-decane and toluene. Specifically, for n-octane, deviations appear at low to moderate densities. in the case of n-nonane, small discrepancies appear at lower $\rho^{*}$ values. On the other hand, for n-decane, the expression fails to accurately capture $D^{*}$ at higher nanochannel widths when the fluid is less dense. As the fluid becomes denser, the region of inaccuracy shifts towards lower channel widths. In the case of toluene, minor errors are present, although they remain within a tolerable range and do not significantly impact the overall accuracy.

## 3. Materials and Methods

In this section, we present the theoretical background for the estimation of the self-diffusion coefficients (Section 3.1), introduce the details of the adopted MD methodology (Section 3.2), perform data preprocessing on the implied dataset (Section 3.3), and briefly describe the SR approach (Section 3.4).

### 3.1. Diffusion Coefficient Calculation

The diffusion coefficient *D* appears in Fick’s first law of diffusion

(6) $$J=-D\cdot \frac{dC}{dx}$$

where *J* is the diffusion flux and $\frac{dC}{dx}$ is the concentration gradient (i.e., the change in concentration over distance). The negative sign indicates diffusion from high to low concentration.

To obtain an accurate *D* value, microscopic-level calculations (such as MD) are usually employed, given either by the Einstein equation

(7) $$D=\lim_{t\to \infty }\frac{1}{2dNt}\langle \sum_{j=1}^{N}{\left[r_{j}\left(t\right)-r_{j}\left(0\right)\right]}^{2}\rangle$$

or the Green–Kubo equation

(8) $$D=\frac{1}{3N}\int_{0}^{\infty }\langle \sum_{j=1}^{N}v_{j}\left(0\right)\cdot v_{j}\left(t\right)\rangle$$

where $r_{j}$ and $v_{j}$ are the position and velocity vectors of the $j^{\mathrm{th}}$ atom, respectively, *N* is the number of atoms, and *d* denotes the dimensionality of the system (*d* = 1 for calculation in one direction, *d* = 2 for two directions, and *d* = 3 for three directions) [38]. Moreover, brackets $\langle \cdot \rangle$ represent the ensemble average over equilibrium trajectories, the quantity $\langle \sum_{j=1}^{N}{\left[r_{j}\left(t\right)\;-\;r_{j}\left(0\right)\right]}^{2}\rangle$ is the mean squared displacement (MSD), and the integrand $\langle \sum_{j=1}^{N}v_{j}\left(0\right)\cdot v_{j}\left(t\right)\rangle$ is the velocity autocorrelation function (VACF). In this paper, the Einstein equation is employed in the MD calculations.

Along with these microscopic-level equations, several mathematical (empirical and/or approximate) relations based on macroscale properties have been proposed, such as the Chapman–Enskog equation [45]:

(9) $$D_{CC}^{*}=\frac{3}{8\sigma^{2}\rho }\sqrt{\frac{k_{B}T^{*}}{m\pi }},$$

where σ is the collision diameter, ρ the number density, *m* the particle mass, and $T^{*}$ the reduced temperature. This equation applies to dilute gases, and its use for liquids requires careful consideration. Speedy et al. [46] proposed an empirical modification to the Chapman–Enskog gas-phase diffusion coefficient $D_{CC}^{*}$ to extend its applicability to higher densities:

(10) $$D_{SP}^{*}=D_{CC}^{*}\left(1-\frac{\rho^{*}}{1.09}\right)\left[1+\rho^{*2}\left(0.4-0.83\rho^{*2}\right)\right]$$

While the Chapman–Enskog formalism and Speedy’s correction describe *D* in dilute to moderately dense systems, a distinct approach is required for liquids and dense fluids where continuum hydrodynamic effects dominate. The Stokes–Einstein equation bridges this gap:

(11) $$D=\frac{k_{B}T}{6\pi \eta R_{h}}$$

where $k_{B}$ is the Boltzmann constant, η is the dynamic viscosity, and $R_{h}$ the hydrodynamic radius of the solute particle.

Zhu et al. [30] suggested a semi-empirical relation for *D* in LJ fluids:

(12) $$D_{LJ}^{*}=\frac{3}{8\sqrt{\pi }}\frac{\sqrt{T^{*}}}{\rho^{*}}A\times B$$

where

$$A=\left(1-\frac{\rho^{*}}{aT^{*b}}\right)\left[1+\rho^{*c}\left(\frac{P_{1}\left(\rho^{*}-1\right)}{P_{2}\left(\rho^{*}-1\right)+T^{*(P_{3}+P_{4}\rho^{*})}}+P_{5}\right)\right]\;\mathrm{and}\;B=e^{-\frac{\rho^{*}}{2T^{*}}}$$

(a, b, c, $P_{1},P_{2},P_{3},P_{4}$, and $P_{5}$ are a set of constants).

It is of interest to note that simpler expressions with similar accuracy have also been derived with SR in Papastamatiou et al. [11] in the form

(13) $$D_{LJ}^{*}=a\cdot \frac{T^{*}}{\rho^{*}}$$

where α is a constant.

### 3.2. Molecular Dynamics

In MD simulations, a particle can act both as a material point and as an approximation point, meaning that the particle can be considered as a single atom or molecule. There, by applying Newton’s law of motion into a system of atoms, the microscopic interactions of a fluid can be simulated. These kinds of intermolecular forces result in different particle positions or velocities, among others, which are being stored during the simulation and further analyzed by quantum mechanic expressions that can approximate the behavior of a physical quantity, such as the diffusion coefficient.

Moreover, interaction-based methodologies are influenced by the model used to define them. A widely recognized particle interatomic potential is the Lennard-Jones (LJ) potential. The 12-6 LJ potential is given by [1]

(14) $$U_{LJ}=4\epsilon \left[{\left(\frac{\sigma }{r}\right)}^{12}-{\left(\frac{\sigma }{r}\right)}^{6}\right]\;,$$

with *r* denoting the particle distance, σ is the interatomic separation where the potential energy is zero, and ϵ is the depth of the potential well. Taking a step beyond simpler potentials (e.g., hard-sphere potential [47]), the LJ potential is capable of effectively modeling more complex frameworks in real fluids [48], especially when approaching dense liquid states [49]. By further selecting to operate in dimensionless LJ units [50], the entire framework becomes computationally efficient and straightforward.

**Figure 8:**
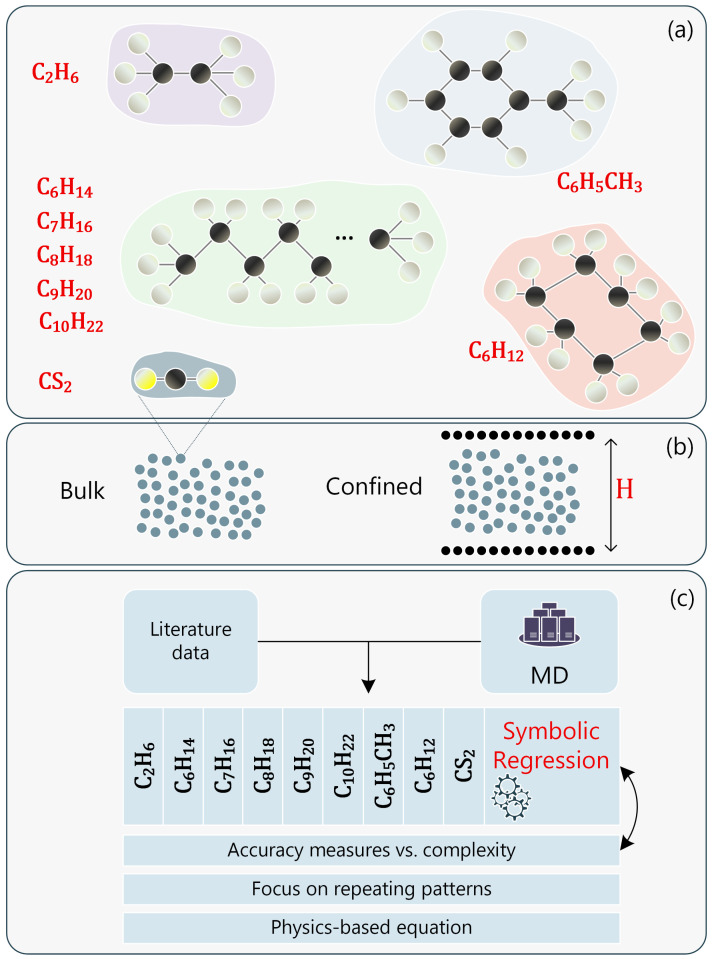
The MD framework consists of (**a**) 9 different molecules (each molecule is a CG bead). (**b**) Simulations are performed for bulk and confined (nanochannel) geometries. (**c**) The SR predictions apply both to each independent molecule and all molecules by considering a universal approach.

For molecular fluids, coarse-grained (CG) methods can also be employed [51]. Every individual molecule is mapped onto a single spherical bead whose non-bonded interactions are described by the LJ potential and can reproduce key structural and dynamical properties of the underlying all-atom system (Figure 8a). For each molecule, a single CG bead is placed at the center of mass of the constituent atoms, with the mass of the CG bead set to the total mass of the mapped atoms, while σ and ϵ are calculated from the Lorentz–Berthelot rule [38]. This approach has been reported in Leverant et al. [35], and it was found to effectively approach the diffusion behavior of CG fluid molecules. This model was tested across various temperatures to confirm that it reproduces liquid-state behavior without un-physical aggregation or crystallization. However, we have to keep in mind that there may be loss of atomistic detail with this approach (i.e., for hydrogen-bonding simulations, polymer melts investigation, and high-density or polar systems) since intramolecular vibrations and specific interactions are averaged out.

Here, we have simulated various CG liquids both at bulk state and nanoconfined structures (Figure 8b). The bulk liquid is in a cubic simulation box with periodic boundary conditions, while the nanoconfined liquids lie between rigid carbon plates. The simulation involves a high-temperature equilibration step running on NVT ensemble with a Langevin thermostat for 100 ps, a cooling phase for 100 ps, and a production run for 1 ns at the target temperature. The time step is set to dt=1 fs, and temperature is controlled by a Nosé–Hoover thermostat with a 100 fs relaxation time. Global momentum is removed every 500 steps to avoid drift. Statistical uncertainties are estimated by block averaging over trajectory segments.

The adopted CG MD framework relies exclusively on the tunable σ, ϵ and *m* parameters, and it is transferable to other non-polar or weakly polar molecular fluids. For a new fluid, these three quantities are refitted, while the simulation pipeline is retained as is. An example MD code can be found in [35].

### 3.3. Data Analysis and Preprocessing

The self-diffusion coefficient of nine molecular fluids, i.e., carbon disulfide, cyclohexane, ethane, n-hexane, n-heptane, n-octane, n-nonane, n-decane, and toluene, has been calculated through MD simulations, and a database has been created for further analysis. For bulk fluids, *D* depends on *T* and ρ, while, for nanochannels, the separation distance *H* is further considered. An initial database has been incorporated from Leverant et al. [35] and subsequently enriched through our own simulations. To ensure consistency and generalization ability of the ML model, all input variables are non-dimensionalized in reduced LJ units as [50]

(15) $$\rho^{*}=\rho \;\sigma^{3},\;T^{*}=\frac{k_{B}\;T}{\epsilon },\;H^{*}=\frac{H}{\sigma },\;D^{*}=D\frac{\sqrt{m/\epsilon }}{\sigma }.$$

Expressing physical variables in a dimensionless form is a common practice and widely employed in ML applications for materials science to enforce physical consistency and improve the ability of the models to generalize. The number of datapoints, *N*, and the range of the reduced variables ($\rho^{*}$, $T^{*}$, $H^{*}$, and $D^{*}$) for each molecular fluid are presented in Table 2 and Table 3.

**Figure 9:**
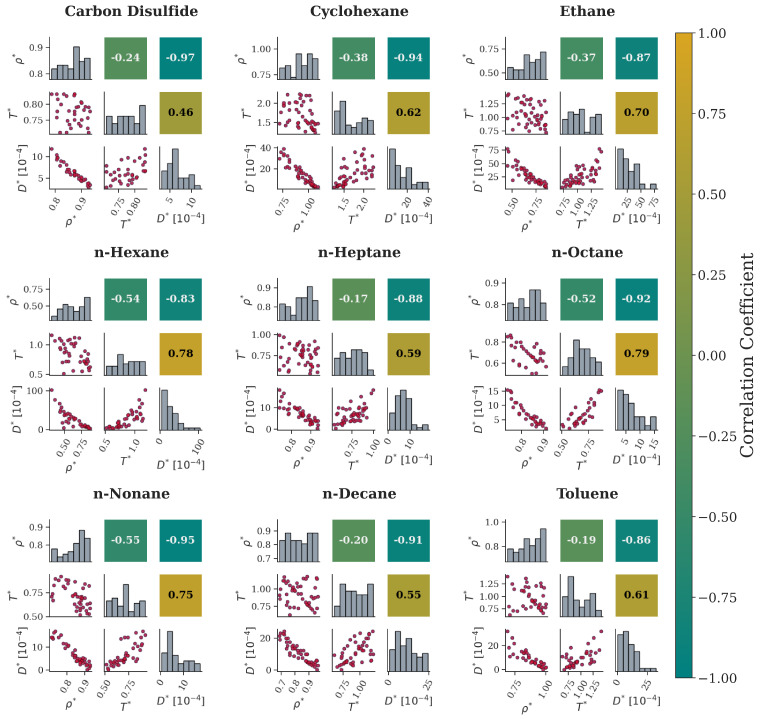
Correlation maps in the form of a matrix for nine molecular fluids at bulk. The lower triangle presents scatterplots of a parameter pair (red dots), the upper triangle (symmetrical to the diagonal) presents the calculated values of the Pearson correlation coefficients, with colors mapped to the colorbar on the right, and the diagonal is the data distribution (grey columns).

**Figure 10:**
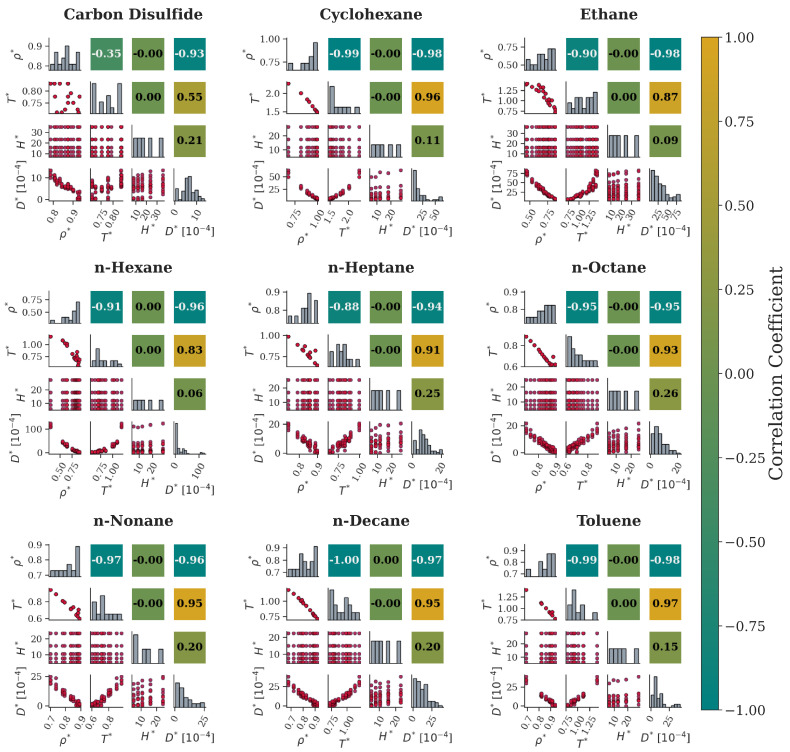
Correlation maps for nine molecular fluids in nanochannels. The lower triangle presents scatterplots of a parameter pair (red dots), the upper triangle (symmetrical to the diagonal) presents the calculated values of the Pearson correlation coefficients, with colors mapped to the colorbar on the right, and the diagonal is the data distribution (grey columns).

To gain qualitative insight regarding the system parameters, correlation maps have been extracted for each fluid. The corresponding scatterplots, histograms, and Pearson correlation values can be found in Figure 9 and Figure 10 for the bulk and nanochannel cases, respectively. For the bulk system (Figure 9), a general trend is observed for $D^{*}$ across all the available fluids. That is, diffusion tends to decrease with $\rho^{*}$ and increase with $T^{*}$. For the $D^{*}-\rho^{*}$ pairs, the relation seems to be linear, as evidenced from the corresponding scatterplots and further supported by the high negative values of Pearson correlation. In contrast, temperature correlation plots suggest that diffusion is increased at higher temperatures as particles may acquire more energy to diffuse. However, their relation is not straightforward, and the Pearson correlation values do not provide additional aid to draw conclusions.

For the nanochannel system (Figure 10), the same qualitative relationship between $\rho^{*}$ and $T^{*}$ is also observed for all the fluids. Moreover, $\rho^{*}$ continues to hinder diffusion since it is seen that $D^{*}$ decreases with the corresponding density increase with an apparently linear relationship, as shown by the scatterplots and Pearson correlation coefficients. Similarly, a temperature increase enhances diffusion in a nearly linear manner. This trend is more evident in most cases except for carbon disulfide and, to a smaller extent, n-hexane. For these two cases, the corresponding scatterplots provide initial evidence that is additionally confirmed by their Pearson values. Finally, the nanochannel width, $H^{*}$, has seemingly no linear correlation with $D^{*}$ for any fluid under consideration, as depicted in their scatterplots. This is also validated by the Pearson values, which indicate the presence of a non-linear correlation.

### 3.4. Symbolic Regression

Symbolic regression is an ML-based method that tries to uncover the governing expression that connects the input parameters with the target quantity. Notwithstanding the fact that plysics-based limitations may be imposed on the process, SR functions even if no prior knowledge about the system exists as it can be fully data-driven. This, in turn, is very helpful in situations where there is no or partial knowledge of the underlying phenomena [52] or for attempting to resolve ambiguous relationships between variables [53], offering more profound solutions. Further insights into SR methods and their applications are available in recent review papers [21,54].

A well-established framework for SR stems from genetic programming (GP) principles [55]. In such a way, the process starts by generating a large set of random symbolic expressions that connect system parameters, represented in a tree-form with nodes and edges (leaves), where the internal nodes can be a mathematical operator or function (e.g., +, −, ×, ÷, pow, exp, log, $\sqrt{x}$, and $x^{c}$), and the terminal nodes of the tree can either be a constant or an input parameter. The most promising components that achieve higher accuracy in terms of an imposed loss function (such as the mean squared error) are iteratively refined through crossover and mutation operations, gradually evolving more effective solutions (see Figure 8c). The main objective is to optimize these expressions by iteratively improving their ability to describe the dataset by minimizing the loss function.

This iterative process is regarded as a multi-objective optimization technique [56] as it can provide multiple solutions that cannot be directly compared to determine a single *best* one. During a common SR implementation, the resulting expressions can range from very simple to more complex. Even more, since GP-based frameworks highly rely on randomness, making them stochastic in nature, the resulting solutions may vary each time the process is run. However, as this GP process iterates in many instances, expressions that keep on appearing, even if they start from a different seed, can be considered as *strong* equations inherent to the available data [11].

The present work builds on the open-access Heuristic Lab software (ver. 3.3) [57] and Python-Julia PySR (ver. 1.5.0) library [58], appropriately embedded in our in-house Python code.

## 4. Conclusions

Symbolic regression and molecular dynamics have been employed in this paper to derive both fluid-specific and all-fluid universal analytical expressions for the self-diffusion coefficient of molecular fluids in bulk and confined channels. Simple yet physically interpretable expressions have been obtained that correlate self-diffusion with macroscopic parameters, such as density, temperature, and channel width, bypassing time- and resource-intensive atomistic simulation methods.

For bulk fluids, all the SR-derived expressions follow a physically consistent inverse proportionality with density and a direct dependence on temperature. A universal all-fluid bulk expression has been also derived, capturing the diffusion trends across nine different molecular fluids with high accuracy. By extending the investigation to nanochannel-confined fluids, a more complex universal equation emerged, incorporating the channel width to account for confinement effects. It is important to highlight that the model predicts the expected physical trend; i.e., self-diffusion decreases under strong confinement and converges to the bulk value as the channel width increases. Moreover, a reduction in self-diffusion due to molecular interactions is captured as molecular mobility increases with temperature.

The universal expressions for bulk and confined systems provide a unified approach applicable to diverse molecular fluids, facilitating the design of nanoscale devices. Future work could explore extending the model to more complex fluids (e.g., mixtures, polar molecules, and ionic liquids) and incorporating additional parameters related to confinement (e.g., the effect of wall materials). Nevertheless, the proposed framework offers a fast, interpretable, and physically consistent tool for predicting self-diffusion in both bulk and nanoconfined environments, which can be easily adjusted to address challenges concerning fluid behavior in engineered systems.

## Figures and Tables

**Table 1:** Values of the $\alpha_{1,2,3,4}$ coefficients that apply to Equation (3) for all molecules at bulk state.

Molecular Fluid	Type	$\alpha_{1}$	$\alpha_{2}$	$\alpha_{3}$	$\alpha_{4}$
Carbon Disulfide	$CS_{2}$	12.83	0.63	2.58	9.507
Cyclohexane	$C_{6}H_{12}$	13.05	0.82	2.59	10.91
Ethane	$C_{2}H_{6}$	22.59	0.91	1.38	15.605
n-Hexane	$C_{6}H_{14}$	23.81	1.26	1.19	12.14
n-Heptane	$C_{7}H_{16}$	12.63	0.68	2.62	9.32
n-Octane	$C_{8}H_{18}$	9.34	0.78	3.17	6.05
n-Nonane	$C_{9}H_{20}$	11.11	0.74	2.84	7.72
n-Decane	$C_{10}H_{22}$	18.84	0.55	1.95	15.605
Toluene	$C_{6}H_{5}CH_{3}$	12.37	0.79	2.55	8.731

**Table 2:** Statistical data analysis for molecular fluids at bulk.

Molecular Fluid	Type	*N*	$\rho^{*}$	$T^{*}$	$D^{*}\;\left[10^{-4}\right]$
Carbon Disulfide	$CS_{2}$	34	0.785–0.932	0.712–0.832	3.015–11.888
Cyclohexane	$C_{6}H_{12}$	49	0.720–1.092	1.253–2.241	2.763–39.433
Ethane	$C_{2}H_{6}$	49	0.449–0.853	0.724–1.426	6.475–77.694
n-Hexane	$C_{6}H_{14}$	40	0.308–0.878	0.516–1.170	3.621–102.942
n-Heptane	$C_{7}H_{16}$	48	0.739–0.937	0.544–0.992	0.551–18.144
n-Octane	$C_{8}H_{18}$	33	0.736–0.911	0.505–0.866	2.107–15.479
n-Nonane	$C_{9}H_{20}$	42	0.715–0.930	0.519–0.919	0.317–17.013
n-Decane	$C_{10}H_{22}$	47	0.687–0.967	0.623–1.186	0.463–24.382
Toluene	$C_{6}H_{5}CH_{3}$	40	0.688–1.001	0.628–1.406	0.102–32.018

**Table 3:** Statistical data analysis for molecular fluids inside nanochannels.

Molecular Fluid	Type	*N*	$\rho^{*}$	$T^{*}$	$H^{*}$	$D^{*}\;\left[10^{-4}\right]$
Carbon Disulfide	$CS_{2}$	80	0.785–0.932	0.712–0.832	7.909–35.590	0.120–13.540
Cyclohexane	$C_{6}H_{12}$	45	0.678–0.995	1.486–2.282	5.933–26.698	5.484–62.824
Ethane	$C_{2}H_{6}$	100	0.449–0.854	0.724–1.426	8.142–36.639	5.747–83.731
n-Hexane	$C_{6}H_{14}$	85	0.308–0.897	0.571–1.170	6.052–27.232	0.002–122.979
n-Heptane	$C_{7}H_{16}$	65	0.739–0.897	0.645–0.992	5.720–25.742	0.331–20.399
n-Octane	$C_{8}H_{18}$	80	0.730–0.896	0.613–0.886	5.508–24.784	0.077–21.614
n-Nonane	$C_{9}H_{20}$	60	0.701–0.891	0.599–0.949	5.287–23.793	0.110–25.000
n-Decane	$C_{10}H_{22}$	75	0.677–0.916	0.720–1.194	5.064–22.788	0.095–37.997
Toluene	$C_{6}H_{5}CH_{3}$	50	0.688–0.939	0.780–1.406	6.233–28.050	0.035–39.326

## Data Availability

Data used in this study and an example symbolic regression code are publicly available at https://github.com/FilSofos/IJMS_Diffusion_Mol_Fluids (accessed on 10 July 2025). MD codes have been adjusted for each molecule from [35].
